# Mechanisms Underlying Reduced Peak Oxygen Uptake in Older Long-Term Breast Cancer Survivors

**DOI:** 10.1016/j.jacadv.2026.102610

**Published:** 2026-03-18

**Authors:** Stephen J. Foulkes, Edith Pituskin, Thomas McMurtry, Rachel J. Skow, Justin Grenier, Nathan Weeldreyer, Corey Tomczak, Deirdre O’Neill, Robert C. Welsh, Richard B. Thompson, Mark J. Haykowsky

**Affiliations:** a Integrated Cardiovascular Exercise Physiology and Rehabilitation Lab, Faculty of Nursing, College of Health Sciences, University of Alberta, Edmonton, Alberta, Canada; b Heart, Exercise and Research Trials Lab, St Vincent’s Institute of Medical Research, Fitzroy, Victoria, Australia; c Department of Radiology and Diagnostic Imaging, Faculty of Medicine and Dentistry, College of Health Sciences, University of Alberta, Edmonton, Alberta, Canada; d Integrative Cardiovascular Physiology Research Program, College of Kinesiology, University of Saskatchewan, Saskatoon, Saskatchewan, Canada; e Division of Cardiology, Faculty of Medicine and Dentistry, College of Health Sciences, University of Alberta, Edmonton, Alberta, Canada; f Hochgebirgsklinik Davos, Medicine Campus Davos, Davos, Switzerland

**Keywords:** cardio-oncology, cardiotoxicity, magnetic resonance imaging, exercise intolerance, cardiorespiratory fitness

## Abstract

**Background:**

Cardiotoxic breast cancer therapy is associated with acute reductions in peak oxygen uptake (VO_2_) (VO_2_peak) that may contribute to future heart failure. Whether impaired VO_2_peak is observed in older long-term breast cancer survivors (BCS) remains unclear.

**Objectives:**

We evaluated VO_2_peak and its Fick determinants in older (≥60 years) long-term female BCS previously treated with cardiotoxic therapy (n = 53; age: 69 ± 5 years; 15 ± 5 years post-therapy) and controls (CON; n = 20; age: 69 ± 5 years).

**Methods:**

Participants underwent maximal cardiopulmonary exercise testing to quantify VO_2_peak. Biventricular ejection fraction, cardiac hemodynamics (cardiac output, stroke volume [SV], and heart rate) and arteriovenous oxygen content difference (C(a-v)O_2_diff) were measured at rest and during submaximal stepping exercise with real-time cardiac and oximetry-based magnetic resonance imaging. Peak calf muscle VO_2_ was calculated from blood flow and C(a-v)O_2_diff, assessed using phase contrast and magnetic resonance imaging oximetry at peak plantar flexion exercise.

**Results:**

Older BCS had lower VO_2_peak than CON (−2.6 mL/kg/min, *P* = 0.019), and a nonsignificantly lower cardiac output augmentation during submaximal stepping exercise (Δ0.6 L/min less, *P* = 0.095), secondary to a blunted augmentation in SV (Δ5 mLs less, *P* = 0.010) and ejection fraction (left-ventricle: Δ3% less, *P* = 0.062; right-ventricle: Δ4% less, *P* = 0.025). There were no group differences in heart rate and C(a-v)O_2_diff during submaximal stepping. Peak calf muscle VO_2_, blood flow, and C(a-v)O_2_diff were also similar between groups.

**Conclusions:**

Compared to CON, older, long-term BCS display lower VO_2_peak, and blunted SV during exertion. The prognostic links between VO_2_peak and future cardiovascular disease suggests decreased VO_2_peak may be an underappreciated risk factor in older long-term BCS.

Breast cancer is the most common malignancy among women worldwide.^1^ Significant improvements in cancer-specific survival, driven by advances in novel therapies, screening, and diagnostics, have resulted in a growing population of breast cancer (BC) survivors (BCS) who are living long-term—defined as more than 5 years postdiagnosis.^1^ However, long-term BCS are increasingly vulnerable to competing negative health effects stemming from cancer treatment and treatment-related comorbidities.^2^ Indeed, cardiovascular disease (CVD) now rivals BC as a major cause of mortality for women diagnosed with early-stage BC.^2^^,^^3^ The risk of CVD are most pronounced for BCS who >60 years^2^, ^3^, ^4^ and/or longer-term survivors (>5-15 years post-BC diagnosis).^3^^,^^4^

Heart failure (HF) is a major driver of cardiovascular morbidity and mortality in older long-term BCS, with an up to 3-fold increased risk of HF hospitalization in older BCS compared to cancer-free older women.^5^ There is increasing recognition that reduced cardiorespiratory fitness, measured as peak oxygen uptake (VO_2_peak), may be an important and underappreciated contributor to elevated risks of CVD, HF, and impaired physical function among BCS.^6^ This concern is supported by well-established associations between low VO_2_peak and an increased risk of HF,^7^ as well mortality rates related to CVD and cancer.^8^^,^^9^ Mounting evidence suggests that adjuvant BC therapy, particularly anthracycline-based chemotherapy, leads to substantial reductions in cardiorespiratory fitness^10^^,^^11^ such that female BCS have VO_2_peak values nearly 30% lower than age-matched women without a history of BC.^12^, ^13^, ^14^ Alternatively, recent observations in older long-term BCS suggest VO_2_peak can be relatively preserved.^15^ Consequently, the clinical implications of such reductions remains poorly understood, in part, due to limited research regarding underlying mechanisms.^6^ Recent studies indicate that VO_2_peak impairments during (neo)adjuvant therapy are also associated with significant declines in peak exercise cardiac output (Qc), stroke volume (SV) and left- and right-ventricular ejection fraction (LVEF and RVEF).^10^^,^^11^^,^^16^^,^^17^ However, other studies have shown skeletal muscle abnormalities, such as capillary rarefaction,^18^ reduced oxidative fibers,^18^ and increased muscle fat infiltration^12^ may also play a role. Moreover, a recent systematic review and meta-analysis of cancer survivors (including breast, colorectal, and esophogastric) exposed to systemic anticancer treatment showed that impairment in arteriovenous oxygen extraction is the predominant factor contributing to reduced VO_2_peak in cancer survivors.^19^ However, these observations are primarily derived from middle-aged cohorts assessed shortly after treatment, a period during which the absolute risk of CVD is relatively low. Given the discrepancy in VO_2_peak impairment noted between younger short-term BCS^20^ and older long-term BCS noted previously,^15^ further research is needed to determine whether these early physiological changes translate into long-term cardiovascular consequences for older BCS—for whom the risk of CVD is more pronounced.

The aim of this study was to assess VO_2_peak (primary outcome) and its Fick determinants (secondary outcomes) during whole body and small muscle mass exercise in long-term BCS ≥60 years compared to age-matched female controls (CON) without a history of BC. We hypothesized that VO_2_peak would be significantly lower in older BCS, attributable to impairments in both central (Qc and SV) and peripheral (arteriovenous oxygen content difference, C(a-v)O_2_diff) Fick determinants.

## Methods

This cross-sectional study combines data from several projects conducted at the University of Alberta between September 2022 and June 2025 that used similar methodologies for the assessment of VO_2_peak, cardiac, and skeletal muscle function in long-term BCS ≥60 years, and healthy older females without BC. Participants completed detailed resting and exercise-based assessments over 2 separate days (within a 2-week period) at the University of Alberta and Mazankowski Alberta Heart Institute. All studies were approved by the Research Ethics Board of the Alberta Cancer Committee (HREBA.CC-22-0254; HREBA.CC-24-0058) and/or the University of Alberta (Pro 00139002), and all participants provided written informed consent before participation.

### Participants and eligibility

#### Breast cancer survivors

Eligibility for BCS included females aged ≥60 years, with a previous diagnosis of early stage (Ia-IIIc) BC, and a history of systemic cardiotoxic therapy (anthracycline-based chemotherapy and/or antihuman epidermal growth factor receptor 2 therapy) completed ≥12 months before enrollment. Participants were recruited from a list of individuals who had participated in previous trials from our group who had consented for future contact, as well as BCS from the Alberta Cancer Research Biobank who met the criteria for the study. Potentially eligible participants were contacted via email or letter by a member of the research team.

#### Controls

Inclusion criteria for the control group included females aged ≥60 years without a history of BC or significant CVD (outlined in the exclusion criteria subsequently). Participants were recruited from a list of those who had previously participated in previous trials from our group, word of mouth, and from advertisements at the University of Alberta Hospital.

#### Screening

Individuals who expressed interest in participating underwent telephone screening by a member of the research team to confirm their eligibility. Treatment and diagnosis details for the BCS group were confirmed directly from their medical records. Exclusion criteria for both groups included a history of 1) coronary artery disease or HF; 2) signs or symptoms of significant myocardial ischemia during exercise testing; 3) significant pulmonary, neurological, or physical impairments precluding exercise assessments; 4) a history of other cancer requiring systemic therapy; and 5) contraindication to magnetic resonance imaging (MRI).

### Cardiopulmonary exercise testing

A detailed description is provided in the Supplemental Methods. In brief, VO_2_peak was assessed from a maximal cardiopulmonary exercise test performed on an upright electromagnetically braked cycle ergometer (Ergoselect 200; ergoline GmbH) with metabolic gas analysis (Vmax Encore Metabolic Cart; SensorMedics Inc; or Parvo TrueOne 2400; Parvo Medics Inc). Heart rate (HR) and rhythm was monitored continuously using 12-lead electrocardiography, with arterial oxygen saturation assessed using finger pulse oximetry. Following a 3-min warm-up at 20 W, participants cycled against increasing resistance using an individualized ramp protocol (10-25 W/min) until volitional fatigue. VO_2_peak was defined as the highest 30 second value obtained during the final 90 seconds of the test from a rolling average of 5 second epochs. VO_2_peak was also expressed as a percentage of age, weight, and sex-predicted according to the Wasserman-Hansen equation.^21^

### Magnetic resonance imaging

On a separate day, participants completed a comprehensive MRI evaluation (Siemens 3T MAGNETOM Prisma; Siemens Healthineers) for the concurrent assessment of VO_2_ and its determinants during submaximal stepping ergometry exercise and incremental-to-maximal plantar flexion exercise. The detailed methodology for the protocol is provided in the Supplemental Methods and Supplemental Figure 1.

#### Stepping exercise cardiac MRI

The stepping exercise evaluation was performed using an MRI-compatible step ergometer (CardioStep; Ergospect). After resting imaging had been completed, participants stepped in time to a metronome at a light-intensity workload (40 steps/min and power output of 20 W), and a moderate intensity workload (60 steps/min and a power output corresponding to 50% of the peak power output obtained during the cardiopulmonary exercise test).

Biventricular volumes and ejection fraction were assessed at rest and during submaximal supine stepping exercise using a validated real-time, free breathing steady-state free precession cine imaging.^11^^,^^22^ In brief, 2 contiguous stacks covering both ventricles in the short axis and long axis views were acquired. Using the short-axis images, endocardial contours were drawn for each ventricle at end-expiration in end-diastole and end-systole by a single experienced investigator (S.J.F.) using custom in-house MATLAB software (RightVol; KU Leuven).

Immediately following the cardiac acquisitions during the same exercise interval, we performed a previously validated MRI susceptometry-based oximetry approach to derive venous oxygen saturation in the inferior vena cava using magnetic field maps acquired using a real-time multiecho gradient-echo pulse sequence.^23^ In combination with arterial oxygen saturation (from finger pulse oximetry) and hemoglobin concentration (from a finger prick blood sample), arterial and venous oxygen content (calculated as arterial or venous oxygen saturation × 1.34 × Hemoglobin concentration) and C(a-v)O_2_diff were calculated.

#### Plantar flexion MRI

Following ∼10 minutes of rest, participants completed an incremental-to-maximal plantar flexion assessment with their dominant leg using an MRI compatible plantar flexion ergometer. Small-muscle mass exercise was included in the study protocol as it allows for the determination of maximal skeletal muscle capacity without limitations imposed by the heart or oxygen delivery.^24^ Participants performed incremental plantar flexion exercise in time to a metronome (30 repetitions/minute) beginning at 4 W, and increasing by 2 W/min until volitional fatigue. At peak exercise, calf muscle blood flow (phase contrast MRI) and venous oxygen saturation (susceptometry-based oximetry) were measured in the popliteal vein, in a perpendicular slice proximal to the knee. These metrics were combined with arterial oxygen saturation and hemoglobin concentration to calculate peak exercise muscle C(a-v)O_2_diff and peak muscle VO_2_ (mVO_2_) as described previously.

### Demographic and medical history

Height and weight were assessed using a stadiometer and electronic scale, respectively, to calculate body mass index and body surface area. Participant age, medical history, and medications were derived from a standardized questionnaire. The duration of self-reported moderate-to-vigorous intensity physical activity completed over the prior week was derived from an adapted version of the International Physical Activity Questionnaire-Short Form. Details related to BC diagnosis and previous therapy were extracted from participant medical records.

### Statistical analysis

Statistical analysis was performed using IBM SPSS Statistics (version 29). Data are presented using mean ± SD, mean (95% CI), median (25th and 75th percentiles), or count (percentage). For baseline characteristics, continuous variables were compared using independent t-tests or Mann-Whitney U-tests depending on the data distribution (assessed using the Shapiro-Wilk test). Categorical variables were assessed using chi-square or Fisher exact tests. With the exception of stepping exercise MRI responses, group differences in study outcomes were assessed using analysis of covariance with adjustment for age— given it may explain residual outcome variance and improve the precision of the effect size estimates related to the independent variable. Differences in stepping exercise MRI responses were assessed using generalized linear models, with group and exercise intensity (and their interaction) included as fixed effects, and participant included as a random effect. For non-normally distributed models, a gamma distribution with a log link was used. Post hoc tests were performed with Bonferroni correction. Statistical significance was set as *P* < 0.05.

## Results

### Participant characteristics

Fifty-three BCS and 20 CON were recruited and included in this analysis. Characteristics for both groups are summarized in Table 1. Both groups were well matched for age, body mass index, and comorbidities, although CON reported significantly more physical activity per week. The BCS group were evaluated 15 ± 5 years post-therapy, with the primary cardiotoxic exposure being anthracycline-based chemotherapy (80% of cohort) with a cumulative doxorubicin equivalent dose of 255 ± 59 mg/m^2^.

**Table 1:** Demographic and Clinical Characteristics for Older Long-Term Breast Cancer Survivors (BCS) and Healthy Controls (CON)

	BCS (n = 53)	CON (n = 20)	*P* Value	SMD
Age, y	69 ± 5	69 ± 5	0.93	0.02
Weight, kg	71.1 ± 13.0	67.7 ± 14.0	0.32	0.25
BMI, kg/m^2^	27.1 ± 5.2	25.6 ± 4.8	0.26	0.30
BSA, m^2^	1.75 ± 0.15	1.72 ± 0.16	0.39	0.26
Hemoglobin, g/dL	14.1 ± 1.0	13.8 ± 0.9	0.19	0.36
Physical activity, mins/wk	180 [30, 285]	225 [210, 360]	0.033	0.28
Comorbidities				
Hypertension, n (%)	18 (34.0%)	6 (30.0%)	0.66	0.09
Hypercholesterolemia, n (%)	14 (26.4%)	5 (25.0%)	0.82	0.03
Diabetes mellitus, n (%)	4 (7.5%)	1 (5.0%)	>0.99	0.11
Medications				
ACEi/ARB, n (%)	15 (28.3%)	3 (15.0%)	0.25	0.33
CCB, n (%)	2 (3.8%)	1 (5.0%)	>0.99	0.06
Beta blocker, n (%)	1 (1.9%)	0 (0.0%)	>0.99	0.2
Statin, n (%)	12 (22.6%)	2 (10.0%)	0.32	0.35
Hypoglycaemic agent, n (%)	3 (5.7%)	0 (0.0%)	0.55	0.35
BC diagnosis details				
Stage I	11 (20.8%)	–	–	–
Stage II	31 (58.5%)	–	–	–
Stage III	11 (20.8%)	–	–	–
BC treatment details				
Time post-therapy, years	15 ± 5	–	–	–
Anthracycline, n (%)	42 (79.2%)	–	–	–
Doxorubicin, n (%)	21 (39.6%)	–	–	–
Epirubicin, n (%)	21 (39.6%)	–	–	–
DOX equivalent dose, mg/m^2^	255 ± 59	–	–	–
Taxane chemotherapy, n (%)	43 (81.1%)	–	–	–
Platinum chemotherapy, n (%)	10 (18.8%)	–	–	–
Anti-HER2 therapy, n (%)	14 (26.4%)	–	–	–
Radiation Therapy, n (%)	42 (79.2%)	–	–	–
Left-sided radiation, n (%)	18 (34.0%)	–	–	–
Radiation dose, Gy	50 ± 5	–	–	–
SERM, n (%)	36 (67.9%)	–	–	–
Aromatase inhibitor, n (%)	18 (3.0%)	–	–	–

Values are mean ± SD, median [25th, 75th percentile], frequency (%) or standardized mean difference (SMD).

ACE-I = angiotensin converting enzyme inhibitor; ARB = angiotensin receptor blocker; BC = breast cancer; BMI = body mass index; BSA = body surface area; CCB = calcium channel blocker; DOX = doxorubicin; HER2 = human epidermal growth factor receptor 2; SERM = selective estrogen receptor modulator.

### Peak oxygen uptake during maximal upright exercise

All participants met the criteria for peak effort except for 1 BCS participant who was limited due to orthopedic issues and was excluded from analysis of cardiopulmonary exercise test-related variables. Results from the cardiopulmonary exercise test are shown in Table 2. Overall, VO_2_peak was significantly lower in BCS vs CON when indexed to body mass (12% lower, *P* = 0.019) and percent predicted (11% lower, *P* = 0.019). Absolute VO_2_peak was lower (8% lower) but this was not statistically significant (*P* = 0.071). Peak oxygen pulse was also significantly lower, whereas there were no significant differences in peak exercise HR or respiratory exchange ratio. The group differences in VO_2_peak were attenuated and no longer statistically significant after repeating the analysis with adjustment for self-reported physical activity (−2.1 mL/kg/min, *P* = 0.085).

**Table 2:** Cardiopulmonary Exercise Test Results in Older Long-Term Breast Cancer Survivors and Healthy Controls

	BCS	CON	Mean Diff (95% CI) BCS vs CON	*P* Value
VO_2_peak, mL/kg/min	19.2 ± 4.2	21.9 ± 5.5	−2.6 (−4.9, −0.4)	0.019
VO_2_peak, L/min	1.33 ± 0.28	1.44 ± 0.28	−0.11 (−0.24, 0.01)	0.071
VO_2_peak, % predicted	90 ± 19	101 ± 27	11 (−21, −2)	0.019
Peak PO, watts	107 ± 22	120 ± 30	−13 (−24, −3)	0.016
Peak HR, b/min	159 ± 20	153 ± 19	6 (−4, 16)	0.24
Peak HR, % predicted	100 ± 12	96 ± 12	4 (−3, 10)	0.24
O_2_ Pulse, mL/beat	8.5 ± 2.0	9.5 ± 2.0	−1.0 (−2.0, −0.0)	0.049
RER	1.27 ± 0.13	1.31 ± 0.10	−0.04 (−0.10, 0.02)	0.21
Peak Ve, L/min	62.1 ± 15.3	69.9 ± 16.7	−7.8 (−15.4, −0.3)	0.041
Peak SBP, mm Hg	186 ± 22	184 ± 27	2 (−12, 15)	0.83

Data are mean ± SD or mean difference (95% CI) for BCS minus CON. Data were assessed using ANCOVA with adjustment for age.

BCS = breast cancer survivors; CON = healthy controls; HR = heart rate; PO = power output; RER = respiratory exchange ratio; SBP = systolic blood pressure; Ve = minute ventilation; VO_2_peak = peak oxygen uptake.

### Oxygen uptake and its determinants during submaximal supine stepping exercise

**Figure 1:**
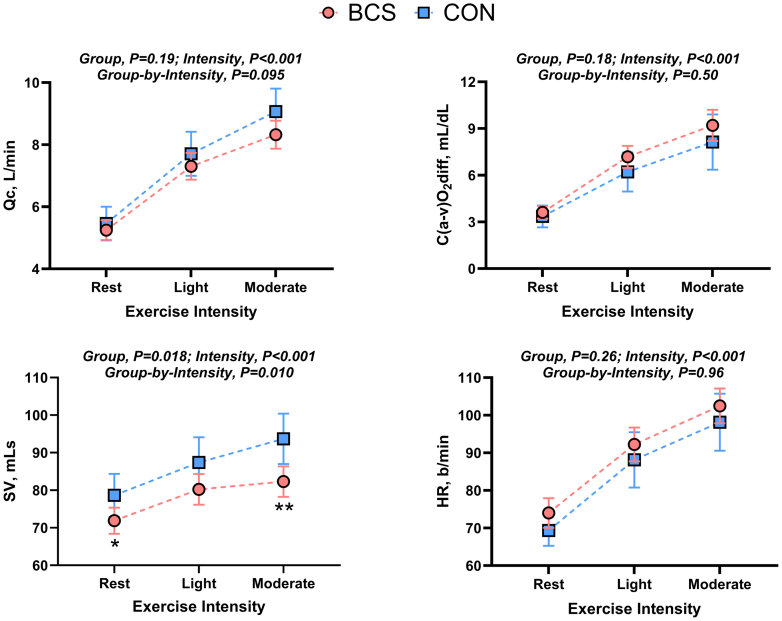
**Fick Determinants of Submaximal Oxygen Intake In Older Long-Term Breast Cancer Survivors and Controls** Cardiac output (Qc), arteriovenous oxygen content difference (C(a-v)O_2_diff), stroke volume (SV), and heart rate (HR) assessed by magnetic resonance imaging at rest and during submaximal stepping exercise in older long-term breast cancer survivors (BCS) and older controls (CON). Data are mean (95% CI), and were compared using generalized linear mixed models with Bonferroni post hoc test. Bonferroni’s post hoc test: ∗*P* < 0.05, ∗∗*P* < 0.01 for BCS vs CON.

**Figure 2:**
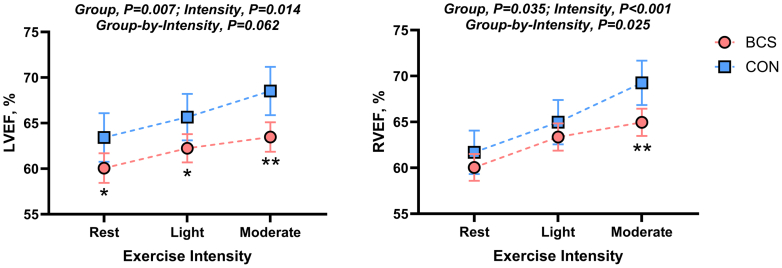
**Biventricular Ejection Fraction Reserve in Older Long-Term Breast Cancer Survivors and Controls** Left- and right-ventricular ejection fraction (LVEF and RVEF) assessed by magnetic resonance imaging at rest and during submaximal stepping exercise in older long-term breast cancer survivors (BCS) and older controls (CON). Data are mean (95% CI), and were compared using generalized linear mixed models with Bonferroni post hoc test. Bonferroni’s post hoc test: ∗*P* < 0.05, ∗∗*P* < 0.01 for breast cancer survivors vs controls.

During submaximal supine stepping exercise MRI assessment, 6 participants could not complete the protocol due to claustrophobia during scanning. Both BCS and CON had similar resting left-ventricular (left-ventricular end-diastolic volume, BCS: 125 ± 22 vs CON: 127 ± 20 mL; *P* = 0.75) and right-ventricular size (right-ventricular end-diastolic volume, BCS: 118 ± 22 vs 125 ± 26 mL; *P* = 0.21). BCS demonstrated significantly impaired SV responses during exercise compared to CON (Figures 1 and 2, with detailed values in Supplemental Table 1). This included both lower resting SV values (72 ± 12 vs 79 ± 13 mL, *P* = 0.049) and significantly blunted augmentation during exercise (ΔSV: 5 mL lower; group-by-intensity interaction, *P* = 0.010), leading to a more pronounced difference in SV at moderate intensity (82 ± 15 vs 94 ± 13 mL, *P* = 0.004). HR responses remained similar between groups with no significant differences or interactions observed. The differences in SV contributed to a numerically smaller Qc augmentation during exercise (ΔQc: 0.6 L/min lower, group-by-intensity interaction, *P* = 0.095) and numerically lower Qc (0.8 L/min lower, *P* = 0.11) at moderate-intensity exercise—but importantly, neither of these observations were statistically significant. Similar results were seen when values indexed to body surface area were evaluated (Supplemental Figure 2).

The smaller SV and blunted SV augmentation may have been the result of a blunted ejection fraction augmentation during exercise in BCS—although this was only statistically significant for RVEF augmentation, whereas LVEF augmentation was nonsignificantly lower (ΔLVEF: 3% less than CON, *P* = 0.062; ΔRVEF: 4% less than CON, *P* = 0.025) (Figure 2). Resting LVEF was significantly lower in BCS (3% lower; *P* = 0.028), and remained lower during light (3% lower; *P* = 0.022) and moderate exercise (5% lower; *P* = 0.001). In contrast, RVEF was not different between BCS and CON at rest and light-intensity exercise, but was significantly lower in BCS at moderate exercise (4% lower; *P* = 0.022).

We were unable to analyze venous oxygen (and subsequently calculate venous oxygen content and C(a-v)O_2_diff) in 5 participants due to excessive participant movement or image artifact. Consequently, 62 participants (BCS: n = 45, CON: n = 17) had data for analysis of venous oxygen saturation and content, and C(a-v)O_2_diff during submaximal stepping exercise (Table 3). Both groups showed a widening of the C(a-v)O_2_diff (BCS: Δ5.6 ± 3.0 mL/dL vs CON: Δ4.8 ± 2.5 mL/dL; intensity *P* < 0.001)) (Figure 1), primarily due to a significant reduction in venous oxygen content (BCS: Δ-5.3 ± 2.4 mL/dL vs CON: −4.8 ± 2.8 mL/dL; intensity *P* < 0.001) and venous oxygen saturation (BCS: Δ-28 ± 13% vs CON: Δ-25 ± 14%; intensity *P* < 0.001). However, there was no group-by-intensity interaction for any of these measures (*P* > 0.21 for all).

**Table 3:** Resting and Exercise Measures of IVC Blood Oxygen Saturation and Content Assessed From Submaximal Stepping Exercise MRI Oximetry in Older Long-Term Breast Cancer Survivors and Healthy Controls

	BCS	CON	Mean Diff (95% CI) BCS vs CON	*P* Value		
				Exercise	Group	Interaction
SaO_2_, %						
Rest	96 ± 2	95 ± 2	1 (0, 2)	0.48	0.85	0.43
Light	95 ± 2	95 ± 2	0 (−1, 1)			
Mod	95 ± 2	95 ± 1	0 (−1, 1)			
SvO_2_, %						
Rest	77 ± 7	77 ± 6	0 (−4, 4)	<0.001	0.54	0.59
Light	58 ± 12∗∗	61 ± 12∗∗	−3 (−10, 4)			
Mod	49 ± 14∗∗∗	52 ± 14∗∗∗	−3 (−11, 6)			
CaO_2_, mL/dL						
Rest	18.1 ± 1.3	17.5 ± 1.3	0.6 (−0.3, 1.4)	0.48	0.15	0.38
Light	17.9 ± 1.3	17.6 ± 1.3	0.3 (−0.4, 1.2)			
Mod	17.9 ± 1.3	17.6 ± 1.2	0.3 (−0.4, 1.1)			
CvO_2_, mL/dL						
Rest	14.6 ± 1.7	14.3 ± 1.5	0.3 (−0.6, 1.4)	<0.001	0.89	0.44
Light	11.0 ± 2.5∗∗	11.4 ± 2.0∗∗	−0.4 (−1.9, 1.0)			
Mod	9.3 ± 2.6∗∗∗	9.5 ± 2.4∗∗∗	−0.2 (−1.7, 1.3)			
C(a-v)O_2_diff, mL/dL						
Rest	3.6 ± 1.3	3.3 ± 1.2	0.3 (2.7, 4.0)	<0.001	0.17	0.45
Light	7.2 ± 2.3∗∗	6.2 ± 2.2∗∗	1.0 (−0.4, 2.4)			
Mod	9.2 ± 3.3∗∗∗	8.1 ± 2.9∗∗∗	1.1 (−0.8, 3.1)			
VO_2_, mL/min^a^						
Rest	187 ± 73	172 ± 90	15 (−32, 62)	<0.001	0.37	0.68
Light	519 ± 209∗∗∗	444 ± 206∗∗∗	75 (−51, 201)			
Mod	767 ± 327∗∗∗	705 ± 352∗∗∗	62 (−139, 263)			

Data are mean ± SD or mean (95% CI). *P* values derived from generalized linear mixed models and 95% CIs (and corresponding post hoc *P* values) corrected for multiple comparisons using Bonferroni post hoc test. Bonferroni’s post hoc test: ∗*P* < 0.05, ∗∗*P* < 0.01 and ∗∗∗*P* < 0.001 for exercise vs rest.

CaO_2_ = arterial oxygen content; C(a-v)O_2_diff = arteriovenous oxygen content difference; CvO_2_ = venous oxygen content; MRI = magnetic resonance imaging; SaO_2_ = arterial oxygen saturation; SvO_2_ = venous oxygen saturation (inferior vena cava [IVC]); VO_2_ = oxygen uptake.

a Calculated in accordance with Fick calculation using cardiac output (Table 3) and C(a-v)O_2_diff.

### Muscle oxygen uptake and its determinants during maximal plantar flexion exercise

**Figure 3:**
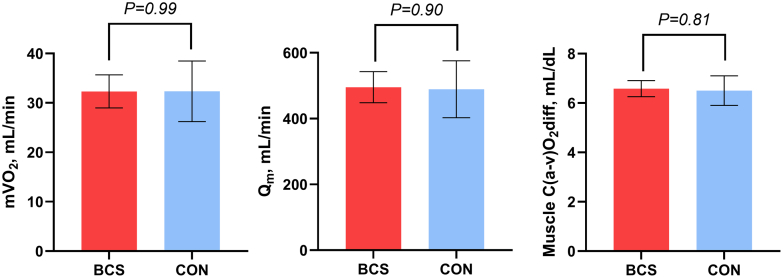
**Peak Calf Muscle Oxygen Intake and Its Fick Determinants in Older Long-Term Breast Cancer Survivors and Controls** Muscle VO_2_ (mVO_2_) and its Fick determinants, including muscle blood flow (Q_m_) and arteriovenous oxygen content difference (C(a-v)O_2_diff) assessed by magnetic resonance imaging at peak plantar flexion exercise in older long-term breast cancer survivors (BCS) and older controls (CON). Data are mean (95% CI) and were assessed using ANCOVA (adjusted for age) with Bonferroni post hoc test. mVO_2_ = muscle oxygen intake.

Peak mVO_2_ and its Fick determinants are shown in Figure 3. Data from the incremental plantar flexion test could not be analyzed in 6 participants due to claustrophobia (n = 3) or poor image quality (n = 3). Both groups showed comparable peak mVO_2_ values, stemming from similar muscle blood flow, venous oxygen saturation, and C(a-v)O_2_diff at peak exercise, with no significant group differences (Figure 3) (*P* > 0.81 for all).

## Discussion

To the best of our knowledge, this is the first study to comprehensively measure VO_2_peak and its determinants during both large submaximal intensity and maximal intensity small muscle mass exercise in older, long-term BCS. Our investigation reveals several new clinically significant findings that advance our understanding of cardiovascular and skeletal muscle health in this population (Central Illustration). The most notable finding is the significantly lower VO_2_peak observed in older BCS compared to CON. Based on our comprehensive cardiac and muscle evaluation, the lower VO_2_peak in BCS is best explained by a blunted SV response to exercise, although this did not result in statistically significant differences in Qc when assessed during moderate intensity exercise—highlighting the need for further exploration of this mechanistic link. Importantly, our maximal muscle exercise MRI evaluations suggest that deficits in skeletal muscle blood flow and oxygenation do not explain the observed VO_2_peak reductions. Overall, our findings provide an additional mechanistic explanation for the greater susceptibility toward CVD and functional disability in older BCS, even in the presence of normal resting left LVEF.

**Central Illustration:**
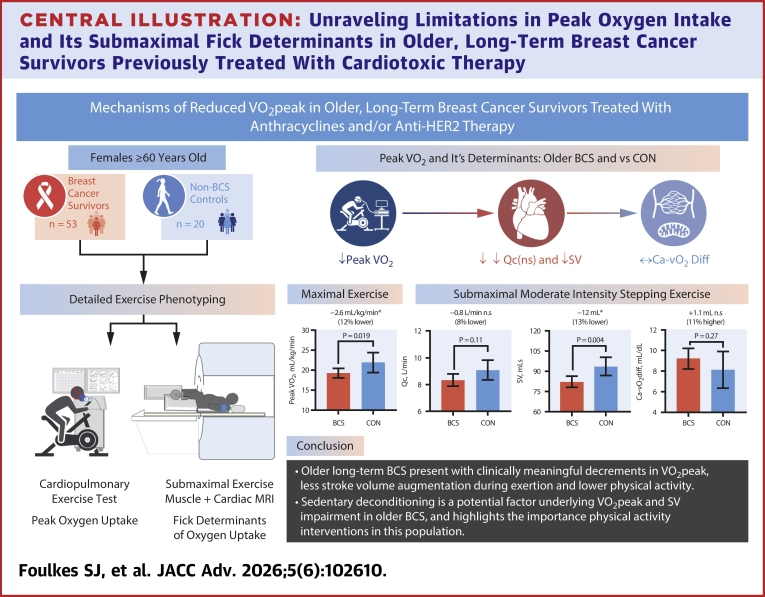
**Unraveling Limitations in Peak Oxygen Intake and Its Submaximal Fick Determinants in Older, Long-Term Breast Cancer Survivors Previously Treated With Cardiotoxic Therapy** Created using BioRender. ∗*P* < 0.05 for BCS vs CON. BCS = breast cancer survivors; C(a-v)O_2_diff = arteriovenous oxygen content difference; CON = controls; HER2 = human epidermal growth factor receptor 2; MRI = magnetic resonance imaging; n.s = not significant for BCS vs CON; Qc = cardiac output; SV = stroke volume; VO_2_ = oxygen intake; VO_2_peak = peak oxygen intake.

### Magnitude of VO_2_peak reductions in long-term BCS and cardiovascular risk implications

Cardiorespiratory fitness is a strong, independent predictor of incident HF,^7^ disability,^25^ and CVD-, and cancer-related mortality.^8^^,^^9^ Therefore, the decrements in VO_2_peak observed in our BCS sample studied on average 15 years post-therapy carry important health implications for older BCS. We found that VO_2_peak was 2.6 mL/kg/min lower (12% lower) in older BCS compared to older CON. This magnitude of reduction is remarkably consistent with decrements measured in BCS <60 years during the course of anthracycline-based (neo)adjuvant therapy, where reductions of 7% to 16% have been reported.^10^^,^^11^^,^^20^ Consequently, middle-aged BCS typically demonstrate VO_2_peak values 17% to 27% lower than age-matched controls.^12^, ^13^, ^14^^,^^20^ Interestingly, percent predicted VO_2_peak in our older BCS was only 10% below predicted values (on average). Whether this represents a regression toward the mean or limitations in prediction equations at the upper extreme of age is an important question. However, despite the lesser magnitude for differences in percent predicted VO_2_peak, as VO_2_peak declines with age, small differences in predicted values can result in more profound implications for daily function, as VO_2_peak (even for the average 70-year-old female with a VO_2_peak of 100% predicted) approaches the threshold required for daily living (ie 18.0 mL/kg/min).^25^

The clinical significance of our findings is apparent by considering that our study cohort was evaluated on average 15 ± 5 years post-therapy, demonstrating that the acute reductions in VO_2_peak observed during the treatment are also observed in BCS evaluated well into long-term survivorship. This is particularly concerning given the established relationships between reduced cardiorespiratory fitness and adverse clinical outcomes. Specifically, the 12% reduction in VO_2_peak translates to ∼13% to 15% increased risk of incident HF, 10% to 12% increased risk of CVD-related mortality, ∼10% to 19% increased risk of cancer-related mortality, 9% to 19% increase in CVD-related, cancer-related, and all-cause mortality, respectively.^7^, ^8^, ^9^ These risk increases are particularly concerning in our cohort of older BCS, as this population already faces heightened cardiovascular risks due to sex- and age-related factors.^2^^,^^4^ The VO_2_peak deficits observed in long-term survivorship may therefore partly explain the well-documented increased risk of CVD in older BCS populations.

Our findings contrast with a recent Scandinavian study of long-term BCS (n = 140, mean age 59 ± 6 years, 11 ± 1 years post-therapy) that found no significant difference in VO_2_peak between BCS (27.6 ± 5.4 mL/kg/min) and CON (27.1 ± 5.4 mL/kg/min).^15^ The reasons for this discrepancy are unclear, and could relate to differences in treatment patterns (eg preferred anthracycline agent and cumulative dose), patient characteristics, or post-treatment lifestyle habits between samples. Indeed, 1 possible explanation for the higher VO_2_peak reported by Saeter et al. may be regional differences in physical activity patterns between their Scadinavian and our North American samples. This suggests that sedentary deconditioning may be an important determinant contributing to the lower VO_2_peak in BCS.^6^^,^^26^ This is partly supported by our observation that adjustment for self-reported physical activity attenuated differences in VO_2_peak between BCS and CON. Furthermore, the strong association between differences in physical activity and VO_2_peak impairment in our BCS sample suggests that lower VO_2_peak in older long-term BCS is due to sedentary deconditioning rather than a direct result of treatment-induced cardiotoxicity. Although, this should be interpreted with caution due to the limitations of subjectively quantifying physical activity via recall with a questionnaire. Regardless, the potential link between physical inactivity and decreased VO_2_peak in BCS should not be ignored, and also speaks to physical activity and/or structured exercise training as potential remedies for addressing VO_2_peak limitations and CVD risk in BCS.^11^

### Exercise-induced cardiac dysfunction and VO_2_peak impairment

Given the established links between VO_2_peak and HF,^7^ there is considerable interest in determining whether cardiac dysfunction is associated with reduced cardiorespiratory fitness in BCS exposed to cardiotoxic therapy.^6^ Our comprehensive exercise cardiac MRI assessments provide unique insights into these mechanisms.

We observed that BCS demonstrated significantly decreased SV augmentation during exercise, which was secondary to lower LVEF and RVEF during moderate intensity exercise. However, the lesser SV did not result in any statistically significant differences in Qc or Qc augmentation at moderate exercise. Therefore, taking the lower SV and biventricular ejection fraction observed during moderate intensity exercise as an explanation for the lower VO_2_peak during maximal upright exercise remains inferential. Nevertheless, SV is considered a major determinant of VO_2_peak across the health and disease spectrum.^27^ Furthermore, the observed differences in SV and numerical differences in Qc between older, long-term BCS and CON provides the most plausible explanation for their lower VO_2_peak—although this mechanistic link requires confirmation by larger, adequately powered studies with simultaneous Qc and VO_2_ measures. These findings generally are consistent with previous studies in BCS evaluated during and shortly following anthracycline-based chemotherapy, which have collectively demonstrated blunted Qc, SV, and/or LVEF responses during exercise using exercise cardiac MRI.^10^^,^^11^^,^^16^ However, using the same gold standard methodology,^22^ we extend these prior findings by demonstrating subclinical impairments in global systolic function (reduced SV and ejection fraction during exertion) are observed in older BCS evaluated more than a decade post-therapy (on average).

Although we cannot definitively establish the exact mechanisms contributing to the observed deficits in SV reserve, several possibilities warrant consideration. The observed impairments may reflect increased myocardial stiffness, altered contractile function, and/or increased afterload during exercise. Kirkham et al.^12^^,^^16^ showed that increased myocardial T1 values, an indirect surrogate for diffuse myocardial fibrosis, were mediators of reduced peak Qc in BCS who were ∼1-year postanthracycline chemotherapy. Furthermore, in a small cohort of older long-term BCS (n = 9, 67 ± 3 years, ∼10 years post-therapy), Beaudry et al.^17^ showed that BCS had a significantly smaller SV at rest and during submaximal exercise, coinciding with increased left-ventricular stiffness—inferred by an increased ratio of left-ventricular end-diastolic volume to E/e’ during exercise. Consequently, further work is needed to understand the physiologic mechanisms contributing to impaired cardiac reserve measured in short- and long-term BCS.

### Peripheral contributions to VO_2_peak limitations

There is growing interest in understanding the potential noncardiac contributions to reduced VO_2_peak in BCS.^6^ Preclinical and human-based studies have demonstrated that anthracycline-based chemotherapy can result in endothelial dysfunction, capillary rarefaction, and significant impairments in skeletal muscle oxidative capacity.^18^^,^^28^^,^^29^ Based on this evidence, we hypothesized that BCS would have a lower C(a-v)O_2_diff during whole body exercise, and a lower calf mVO_2_ and C(a-v)O_2_diff during isolated small muscle exercise. Contrary to our hypothesis, we found comparable values for all of these peripheral measures between BCS and CON groups. This finding is consistent with previous research by Beaudry et al.,^13^ who used the same exercise MRI technique and observed similar mVO_2_ and muscle oxygen extraction during submaximal plantar flexion exercise in middle-aged (mean age 56 years) BCS (n = 16) compared to CON (n = 16).

The longitudinal study of Kirkham et al.^30^ also showed that submaximal mVO_2_ paradoxically increased over the course of adjuvant BC chemotherapy. This increase was attributed to a higher metabolic oxygen cost of plantar flexion exercise, as peak plantar flexion power output remained stable throughout the study period. In contrast, we saw similar peak mVO_2_ and plantar flexion power output values, and similar C(a-v)O_2_diff during submaximal stepping exercise in our cohort measured on average 15 years post-therapy. Taken together, these results suggest impairments in microvascular or skeletal muscle structure/function may not be major drivers of reductions in VO_2_peak in older, long-term BCS. However, caution is warranted, as it is also possible that the muscle mass engaged in plantar flexion exercise was too small for us to unmask appreciable impairments in skeletal muscle function. Moreover, due to the submaximal nature of our whole-body stepping exercise procedure—it is possible that the intensity was insufficient to unmask deficits in peripheral function. Therefore, further work is needed to assess peripheral components of oxygen transport and utilization in BCS using larger muscle mass and/or higher intensity exercise.

It is also possible that body composition abnormalities, such as muscle atrophy, muscle fat infiltration, or excessive global ectopic fat deposition could contribute to VO_2_peak impairment—and may explain why the difference in absolute VO_2_peak between BCS and CON was smaller (and not statistically significant) than when indexed to bodyweight. However, we did not perform body composition assessment to test this potential mechanistic association.

### Clinical implications

The findings from this study highlight a need for strategies to address the effects of BC therapy on VO_2_peak and its determinants in older BCS. Our observation that a large degree of the VO_2_peak decrement in BCS was related to lower physical activity suggests that the lower VO_2_peak and SV in older long-term BCS could potentially reflect reversible cardiac changes due to sedentary deconditioning that could be targeted with exercise or physical activity interventions, rather than direct treatment-induced cardiac injury. Indeed, the BREXIT (Breast Cancer Exercise Intervention) study^11^ showed that structured exercise training during and after anthracycline-based chemotherapy can effectively protect against decreases in VO_2_peak and cardiac reserve in a younger cohort of BCS (mean age 50 years). However, this model may not be possible for all patients—many of whom may not present with cardiovascular issues until older age. A recent study from Johansen et al.^31^ showed that 5 months of aerobic exercise training improves VO_2_peak (+1.2 mL/kg/min) in long-term BCS (n = 140, mean age 59 years, ∼11 years post-therapy); however, the effects were blunted compared to age-matched women without BC (+2.6 mL/kg/min), and were also substantially less than that reported in the younger BREXIT cohort (+3.5 mL/kg/min).^11^ This raises the question of whether the cardiovascular system of older BCS is less adaptable to interventions such as exercise training. However, Johansen et al.^31^ did not assess the determinants of VO_2_peak. Therefore, it is unclear whether exercise training can correct the cardiac limitations present in older BCS, or whether exercise is primarily a strategy to induce compensatory vascular and/or skeletal muscle adaptations. Consequently, the impact of therapies such as exercise training on VO_2_peak and its determinants (including reduced cardiac function) in older BCS is an important question.

### Study Limitations

Most notably, the cross-sectional design precludes attribution of cause and effect to BC therapy and/or sedentary deconditioning. It is possible that factors independent of cardiotoxic therapy could partly or wholly explain the observed deficits in VO_2_peak and cardiac function in BCS. Indeed, based on the multiple-hit hypothesis, CVD in BCS is explained by the contribution of multiple factors (sedentary deconditioning, weight gain, and premature menopause) beyond exposure to cardiotoxic therapy, and our results suggest sedentary deconditioning may be a particularly important factor in this setting. However, these relationships require validation in adequately powered longitudinal studies. The small control group sample (N = 20) likely limited our statistical precision, and may have resulted in insufficient power to detect meaningful differences in Qc and LVEF augmentation that would provide complimentary insights into the observed differences in VO_2_peak and SV. It is also possible that the submaximal nature (stepping exercise) and/or small muscle mass (plantar flexion exercise) used for the exercise MRI assessments was insufficient to properly unmask statistically significant differences between groups. Indeed, this may be why we only saw a statistical trend for lesser Qc augmentation in BCS, whereas other studies evaluating peak exercise Qc have shown significant effects.^10^^,^^12^ Our results could also be affected by enrollment and survivor biases due to the long period between BC diagnosis and enrollment, the focus on exercise-based assessments (potentially favoring fitter BCS) and exclusion of BCS with overt CVD. We also did not exclude participants who were meeting or exceeding physical activity guidelines, and in fact, the median self-reported physical activity levels classify both groups as sufficiently active. These criteria may have excluded BCS with more overt cardiotoxicity, and led to a healthier-than-expected BCS sample—limiting generalizability of our findings to patients who have overt cardiotoxicity or who are predominantly sedentary. However, these factors bias toward the null hypothesis, rather than magnifying the differences we have measured.

## Conclusions

Older, long-term BCS have reduced VO_2_peak, which coincided with lesser SV augmentation and lower ejection fraction during moderate intensity stepping exercise, whereas skeletal muscle function was largely preserved. Given the increased risk of CVD in older BCS, this raises the question of whether decreased VO_2_peak and blunted SV reserve could be underappreciated factors contributing to functional impairment and CVD in BCS. The link between physical activity and VO_2_peak decrement in older BCS suggests that sedentary deconditioning is a potential factor underlying VO_2_peak impairment in older BCS, and highlights the importance physical activity interventions in this population.

Perspectives **COMPETENCY IN MEDICAL KNOWLEDGE:** Older long-term BCS with preserved resting LVEF present with clinically meaningful decrements in VO_2_peak that coincided with impairments in SV augmentation during exertion and decreased levels of physical activity. This highlights fitness and cardiac function impairments are present even in older BCS measured many years after therapy, and provides a rationale for pharmacologic or lifestyle intervention to address these cardiovascular and functional impairments across the survivorship continuum.

**TRANSLATIONAL OUTLOOK:** Decreased VO_2_peak may be an underappreciated risk factor for future CVD in older BCS; however, further work is needed to understand the prognostic importance of decreased VO_2_peak for future HF in this population.

## Funding support and author disclosures

This work was partly supported by a Project Grant from the 10.13039/501100000024Canadian Institutes of Health Research (CIHR; PJT 183969), and an Operating Grant from the 10.13039/100009326Cancer Research Society (1280836) / 10.13039/501100000024CIHR – Institute for cancer research (196960). Dr Foulkes is supported by a fellowship from the L.E.W Carty Charitable Fund. All other authors have reported that they have no relationships relevant to the contents of this paper to disclose.
