# Supplementary material for: Mechanisms Underlying Reduced Peak Oxygen Uptake in Older Long-Term Breast Cancer Survivors
Source: JACC Adv. 2026 Mar 18;5(6):102610. doi: 10.1016/j.jacadv.2026.102610 (PMC13309329; doi:10.1016/j.jacadv.2026.102610)
Supplement: Supplemental Material [file mmc1.docx]

**Supplementary Material**

**Mechanisms underlying reduced peak oxygen uptake in**

**older long-term breast cancer survivors**

Stephen J Foulkes,^1,2*^ Edith Pituskin,^1*^ Thomas McMurtry,^1^ Rachel J Skow,^1^

Justin Grenier,^3^ Nathan Weeldreyer,^1^ Corey Tomczak,^4^ Deirdre O’Neill,^5^

Robert C. Welsh,^5^ Richard B Thompson,^3#^ Mark J Haykowsky^1,5#^

* Contributed equally as first-authors, ^#^ contributed equally as senior authors

^1^ Integrated Cardiovascular Exercise Physiology and Rehabilitation Lab, Faculty of Nursing, College of Health Sciences, University of Alberta, Edmonton, AB, Canada

^2^ Heart, Exercise and Research Trials Lab, St Vincent’s Institute of Medical Research, Fitzroy, VIC, Australia

^3^ Department of Radiology and Diagnostic Imaging, Faculty of Medicine and Dentistry, College of Health Sciences, University of Alberta, Edmonton, AB, Canada

^4^ Integrative Cardiovascular Physiology Research Program, College of Kinesiology, University of Saskatchewan, Saskatoon, SK, Canada

^5^ Division of Cardiology, Faculty of Medicine and Dentistry, College of Health Sciences, University of Alberta, Edmonton, AB, Canada

^5^Hochgebirgsklinik Davos, Medicine Campus Davos, Davos, Switzerland.

**Supplementary Methods**

***Cardiopulmonary Exercise Testing***

A maximal cardiopulmonary exercise test (CPET) was performed on an upright electromagnetically braked cycle ergometer (Ergoselect 200, ergoline GmbH, Germany) with metabolic gas analysis (Vmax® Encore Metabolic Cart, SensorMedics Inc., USA; or Parvo TrueOne 2400, Parvo Medics Inc., USA) for the assessment of peak oxygen uptake (VO_2_peak). Heart rate (HR) and rhythm was monitored continuously using 12-lead electrocardiography, with arterial oxygen saturation (SaO_2_) assessed using finger pulse oximetry. Brachial blood pressure was assessed at rest and every two minutes during the test. Following three minutes of rest, participants completed a three-minute warm-up at 20 watts, followed by a ramp protocol with increments of 10-25 watts/minute (individualized to participant age, sex, body size and physical activity history) such that they would reach volitional exhaustion within 8-12 minutes. Participants were instructed to maintain their cadence at >70 revolutions per minute for the duration of the test. VO_2_peak was defined as the highest 30 second value obtained during the final 90 seconds of the test from a rolling average of five second epochs. Criteria for peak effort included achieving three out of i) a plateau in VO_2_ despite increasing workload; ii) an inability to maintain cadence >60 revolutions per minute; iii) HR ≥85% of age-predicted values according to the Tanaka et al. formula^26^; iv) a peak respiratory exchange ratio >1.10 and/or v) a maximal rating of perceived exertion ≥7 out of 10 (indicative of very hard subjective effort).

***Magnetic Resonance Imaging***

On a separate day, participants completed a comprehensive magnetic resonance imaging (MRI) evaluation (Siemens 3T MAGNETOM Prisma, Siemens Healthineers, Germany) for the concurrent assessment of VO_2_ and its determinants during submaximal stepping ergometry exercise and incremental-to-maximal plantar flexion exercise (Figure 1).

*Stepping Exercise Cardiac MRI (ExCMR):* The stepping exercise evaluation was performed using an MRI-compatible step ergometer (CardioStep, Ergospect, Austria). After resting imaging had been completed, participants were instructed to begin stepping in time to a metronome at a tempo of 40 steps/min (light intensity stage) and 60 steps/min (moderate intensity stage). Participants completed two workloads, including a standardised light-intensity workload at 20 watts, and a moderate intensity workload corresponding to 50% of the peak power output obtained during the CPET from their first study visit. Imaging was performed after participants had been exercising at each workload for at least two minutes to ensure a hemodynamic steady-state had been achieved, such that participants performed each workload for ~5-minutes.

Bi-ventricular cardiac volumes and ejection fraction (EF) were assessed at rest and during submaximal supine stepping exercise using real-time, free breathing steady-state free precession cine imaging. This approach has been previously described in detail^18,27^ and validated against right-heart catheterisation.^27^ In brief, two contiguous stacks covering both ventricles in the short axis and long axis views were acquired (Supplementary Figure 1A). Using the short-axis images, endocardial contours were drawn for each ventricle at end-expiration in end-diastole and end-systole by a single experienced investigator (SJF) using custom in-house MATLAB software (RightVol, KU Leuven, Belgium). The long-axis images were used as reference images to ensure accurate contouring of the atrio-ventricular plane. Stroke volume (SV) for the left- and right-ventricle was calculated as the difference between end-diastolic and end-systolic volume, and EF (LVEF, RVEF) was calculated as the ratio of SV to end-diastolic volume. Cardiac output was calculated as ([right ventricular SV + left-ventricular SV] ÷ 2) x HR.

Immediately following the cardiac acquisitions during the same exercise interval, we performed a previously validated MRI susceptometry-based oximetry approach to derive venous oxygen saturation (SvO_2_) in the inferior vena cava (Supplementary Figure 1B).^28^ As previously described,^28,29^ SvO_2_ was measured from magnetic field maps acquired using a real-time multi-echo gradient-echo pulse sequence applied over a 30 second interval immediately following cardiac imaging (296 ms sampling interval, 100 acquired images). Retrospective selection of images at a consistent respiratory phase minimized motion artifacts.^28^ In combination with SaO_2_ (measured continuously using finger pulse oximetry) and hemoglobin concentration (Hb, measured via a finger prick blood sample; HemoCue Hb 201+, HemoCue, Sweden) arterial and venous oxygen content (CaO_2_, CvO_2_, calculated as SaO_2_ or SvO_2_ x 1.34 x Hb) and the arterio-venous oxygen content difference (C(a-v)O_2_diff) were calculated. Oxygen uptake was then calculated as [Qc x C(a-v)O_2_diff] in accordance with the Fick principle. We have previously shown excellent agreement (R^2^=0.94) between VO_2_ derived from this MRI approach and via metabolic gas analysis.^28^

*Plantar Flexion MRI:* Following ~10 minutes of rest, participants then completed an incremental-to-maximal plantar flexion assessment with their dominant leg using an MRI compatible plantar flexion ergometer (TriSpect, Ergospect, Austria) to derive peak calf muscle VO_2_ (mVO_2_), blood flow, SvO_2_ and C(a-v)O_2_diff. Small-muscle mass exercise was included in the study protocol as it allows for the determination of maximal skeletal muscle function without limitations imposed by the heart.^30^ The methodology is similar to that previously developed and described by our group.^21,29,31^ In brief, after acquiring resting images, participants performed plantar flexion exercise in time to a metronome (30 repetitions per minute) beginning at 4 watts, and increasing by 2 watts/minute until volitional exhaustion. Within ≤1 second of completing exercise, calf muscle blood flow (phase contrast MRI) and SvO_2_ (susceptometry-based oximetry) were measured in the popliteal vein, in a perpendicular slice proximal to the knee (Supplementary Figure 1C). These metrics were combined with SaO_2_ and Hb to calculate peak exercise muscle C(a-v)O_2_diff and mVO_2_ using the same calculations as described above (except muscle blood flow is used in place of Qc).

| **Supplementary Table 1.** Resting and exercise cardiac function assessed from submaximal stepping exercise cardiac MRI in older long-term breast cancer survivors (BCS) and healthy controls (CON). | | | | | | |
| --- | --- | --- | --- | --- | --- | --- |
| **Outcome** | **BCS** | **CON** | **Mean Diff (95% CI)**  **BCS vs CON** | **Exercise**  **P** | **Group**  **P** | **Interaction**  **P** |
| ***Central Hemodynamics*** | | | | | | |
| *Qc, L/min* |  |  |  | <0.001 | 0.19 | 0.095 |
| Rest | 5.3±1.0 | 5.5±1.3 | -0.2 (-0.8, 0.4) |  |  |  |
| Light | 7.3±1.4*** | 7.7±1.8*** | -0.4 (-1.2, 0.4) |  |  |  |
| Mod | 8.3±1.5*** | 9.1±1.7*** | -0.8 (-1.6, 0.9) |  |  |  |
| *SV, mL* |  |  |  | <0.001 | 0.018 | 0.010 |
| Rest | 72±12 | 79±13 | -7 (-13, 0)^†^ |  |  |  |
| Light | 80±15*** | 87±12*** | -7 (-15, 1) |  |  |  |
| Mod | 82±15*** | 94±13*** | -12 (-19, -4)^††^ |  |  |  |
| *HR, b/min* |  |  |  | <0.001 | 0.26 | 0.96 |
| Rest | 74±14 | 69±12 | 5 (-3, 12) |  |  |  |
| Light | 92±16*** | 88±14*** | 4 (-4, 12) |  |  |  |
| Mod | 102±17*** | 98±11*** | 4 (-4, 13) |  |  |  |
| ***Ejection Fraction*** | | | | | | |
| *LVEF, %* |  |  |  | <0.001 | 0.007 | 0.062 |
| Rest | 60±6 | 63±4 | -3 (-6, 0)^†^ |  |  |  |
| Light | 62±6** | 66±4** | -3 (-6, -1)^†^ |  |  |  |
| Mod | 63±6*** | 69±4*** | -6 (-9, -2)^††^ |  |  |  |
| *RVEF, %* |  |  |  | <0.001 | 0.034 | 0.025 |
| Rest | 60±5 | 62±5 | -2 (-5, 1) |  |  |  |
| Light | 63±5*** | 65±5** | -2 (-4, 1) |  |  |  |
| Mod | 65±5*** | 69±4*** | -4 (-7, -2)^†^ |  |  |  |
| Data are mean±SD or mean (95% CI). P-values derived from generalized linear mixed models, and 95% CI’s corrected for multiple comparisons using Bonferroni post-hoc method. Bonferroni corrected Post-hoc: * P<0.05, ** P<0.01 and *** P<-0.001 for exercise vs rest; ^†^ P<0.05, and ^††^ P<0.01 for BCS vs CON. Abbreviations: Qc, cardiac output; SV, stroke volume; HR, heart rate; LVEF, left-ventricular ejection fraction; RVEF, right-ventricular ejection fraction. | | | | | | |


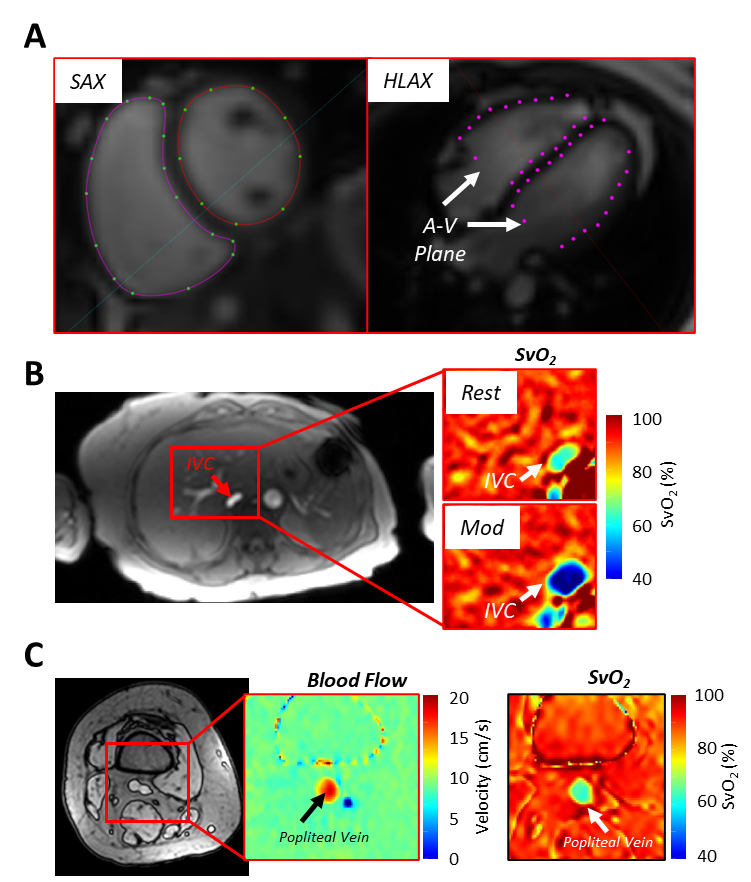


**Supplementary Figure 1. Methodology for deriving oxygen uptake and its Fick determinants using exercise Magnetic Resonance Imaging.** (A) A contiguous stack covering both ventricles in the short-axis [SAX] and horizontal long-axis [HLAX] view were acquired using real-time free breathing steady-state free precession imaging at rest and during submaximal stepping exercise. As shown in the case example, endocardial contours were drawn on the SAX images at end-expiration during end-diastole and end-systole to derive biventricular volumes and cardiac output, with reference to the HLAX images to ensure accurate contouring of the atrio-ventricular [A-V] plane. (B) Immediately following cardiac imaging (and during the same exercise bout) susceptometry-based oximetry images were acquired of the inferior vena cava [IVC] adjacent to the liver. The ferromagnetic field shift caused by deoxyhemoglobin in the IVC relative to surrounding liver tissue was used to calculate venous oxygen saturation [SvO_2_] for Fick calculations. A representative example of SvO_2_ measured at rest and during moderate intensity stepping is shown. (C) Illustrative images taken immediately following maximal plantar flexion exercise demonstrate how a combination of phase contrast imaging and susceptometry-based oximetry were used to derive blood flow velocity and SvO_2_, respectively, in the popliteal vein.

**Supplementary Figure 2. Rest and exercise cardiac haemodynamics in older long-term BCS and CON indexed to body surface area.** Comparison of cardiac index (CI) and stroke volume index (SVi) assessed by magnetic resonance imaging at rest and during submaximal stepping exercise in older long-term breast cancer survivors (BCS) and older controls (CON). Data are mean (95%CI), and were compared using generalized linear mixed models with Bonferroni post-hoc test. Post-hoc: * P<0.05, ** P<0.01 for BCS vs CON.
